# The complete chloroplast genome and phylogenetic analysis of *Veratrum mengtzeanum* Loes. F. (Liliaceae)

**DOI:** 10.1080/23802359.2019.1693926

**Published:** 2019-11-20

**Authors:** Li-Jun Han, Ying-Ying Liu, Ying-Min Zhang, Cong-Wei Yang, Zi-Gang Qian, Guo-Dong Li

**Affiliations:** a Faculty of Traditional Chinese Pharmacy, Yunnan University of Chinese Medicine, Kunming, China;; b Yunnan Insitute for Food and Drug, Kunming, China;; c Yunnan Key Laboratory for Dai and Yi Medicines, Yunnan University of Chinese Medicine, Kunming, China

**Keywords:** Veratrum mengtzeanum, chloroplast genome, Liliaceae, phylogenetic analysis

## Abstract

*Veratrum mengtzeanum* Loes. F. is a medicinal plant belonging to the genus *Veratrum* (Liliaceae). In the present study, we assembled and characterized the complete chloroplast (cp) genome of this species. The chloroplast genome is 152,051 bp in length, with one large single copy (LSC) region and one small single copy (SSC) region of 82,112 bp and 17,544 bp, respectively; two inverted repeat (IR) regions of 26,198 bp. It contains 131 annotated genes, including 85 protein-coding genes (PCGs), 38 transfer RNA (tRNA) genes, and 8 ribosomal RNA (rRNA) genes. Phylogenetic analysis indicated that *V. mengtzeanum* was closely related to *Veratrum japonicum* with 100% bootstrap value.

*Veratrum mengtzeanum* Loes. F., a perennial herb, endemic to southwest China, is a medicinal and poisonous plant belonging to the genus *Veratrum* (Liliaceae). It grows under the forest or beside the hill sided-road with altitude of 1200–3200 m (Yin et al. 2014). There are about 43 species of the genus *Veratrum* in the world (Gu et al. 2018), most of which are used as the medicinal plants (Yin et al. 2016). The roots and rhizomes of *V. mengtzeanum* have been used as traditional folk medicine named “Pimacao” in Yunnan province, used for treating traumatic injuries, fracture, paraplegia, epilepsy, rheumatic pain and traumatic hemorrhage (Li et al. 2019). It is the main component of the renowned traditional Chinese medicine, “YunnanBaiyao”. To date no study has been carried out on the genome of *V. mengtzeanum*. To provide a rich genetic information and improve *V. mengtzeanum* molecular breeding in the future, we report and characterize the complete chloroplast genome sequence of *V. mengtzeanum* (GenBank accession number: MN589932).

Fresh leaves of *V. mengtzeanum* were collected from Gejiu (N23°21′, E103°10′), Honghe Hani and Yi Autonomous Prefecture of Yunnan, China and voucher specimens (5325030256) were deposited in Herbarium of Yunnan University of Chinese Medicine. Firstly, the genomic DNA was extracted using the plant DNA extraction kit (Bioteke Corporation, China). Then genome sequencing was performed on the Illumina Hiseq 2500 platform with a library construction. Secondly, we de novo trimmed and assembled the raw data of 3 Gb based on NOVOPlasty (Dierckxsens et al. 2017). And the complete cp genome we obtained was annotated with the online annotation tool GeSeq (Tillich et al. 2017).

The complete cp genome of *V. mengtzeanum* is 152,051 bp, including a large single copy (LSC) region of 82,112 bp and a small single copy (SSC) region of 17,544 bp, separated by a pair of inverted repeat (IR) regions of 26,198 bp. The overall GC content of the whole plastome, LSC, SSC and IR regions are 37.8%, 35.8%, 31.5%, 42.9%, respectively. The cp genome has 131 annotated genes, including 85 protein-coding genes, 38 tRNA genes, and eight rRNA genes. A total of 64 SSRs were detected using the online software IMEx (Mudunuri and Nagarajaram 2007). The number of mono-, di-, tri-, tetra-, penta-, and hexa-nucleotides SSRs are 44, 11, 3, 5, 1, and 0, respectively.

To further infer the phylogenetic position of *V. mengtzeanum*, plastome of 25 representative species were obtained from NCBI to construct the plastome phylogeny, with two species of Araceae and Colocasia as outgroups. All the plastomes were aligned using MAFFT v.7 (Katoh and Standley. 2013). And we used the RAxML (Stamatakis 2014) with 1000 bootstrap replicates for supporting the branches evaluated under the GTR model. The phylogenetic tree shows that *V. mengtzeanum* was closer to *V. japonicum* compared with other species (Figure 1).

**Figure 1.**
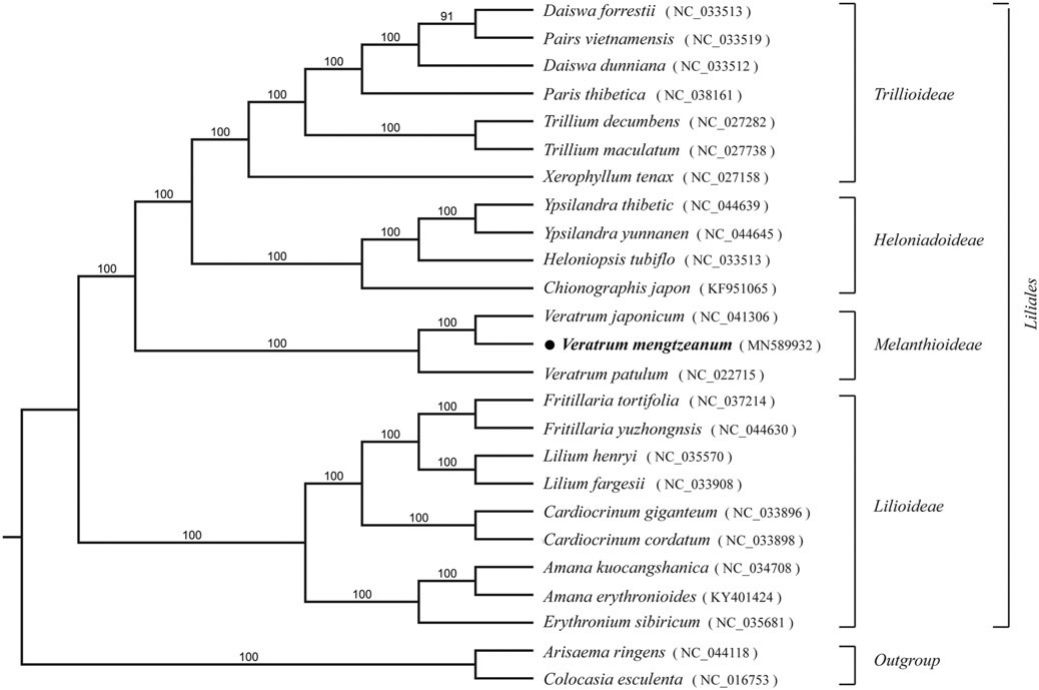
The Maximum likelihood phylogenetic tree inferred from 25 chloroplast genomes. Bootstrap support values >50% are indicated next to the branches. GenBank accession numbers are given in figure.

In summary, the complete cp genome from this study provides significant insight for elucidating the phylogenetic relationship of taxa genus *Veratrum*.
